# In-Situ Synthesis and Characterization of Chitosan/Hydroxyapatite Nanocomposite Coatings to Improve the Bioactive Properties of Ti6Al4V Substrates

**DOI:** 10.3390/ma13173772

**Published:** 2020-08-26

**Authors:** Zahra Ansari, Mahdi Kalantar, Alessandra Soriente, Ines Fasolino, Mahshid Kharaziha, Luigi Ambrosio, Maria Grazia Raucci

**Affiliations:** 1 Department of Mining and Metallurgical Engineering, Yazd University, Yazd 89195-741, Iran; z.ansari64@gmail.com; 2 Institute of Polymers, Composites and Biomaterials, National Research Council (IPCB-CNR), 80078 Naples, Italy; alessandra.soriente@cnr.it (A.S.); ines.fasolino@cnr.it (I.F.); luigi.ambrosio@cnr.it (L.A.); 3 Biomaterials Research Group, Department of Materials Engineering, Isfahan University of Technology, Isfahan 84156-83111, Iran; kharaziha@cc.iut.ac.ir

**Keywords:** Ti6Al4V alloy, chitosan, hydroxyapatite, nanocomposite coatings, sol-gel method, in vitro bioactivity

## Abstract

Ti6Al4V alloy is still attracting great interest because of its application as an implant material for hard tissue repair. This research aims to produce and investigate in-situ chitosan/hydroxyapatite (CS/HA) nanocomposite coatings based on different amounts of HA (10, 50 and 60 wt.%) on alkali-treated Ti6Al4V substrate through the sol-gel process to enhance in vitro bioactivity. The influence of different contents of HA on the morphology, contact angle, roughness, adhesion strength, and in vitro bioactivity of the CS/HA coatings was studied. Results confirmed that, with increasing the HA content, the surface morphology of crack-free CS/HA coatings changed for nucleation modification and HA nanocrystals growth, and consequently, the surface roughness of the coatings increased. Furthermore, the bioactivity of the CS/HA nanocomposite coatings enhanced bone-like apatite layer formation on the material surface with increasing HA content. Moreover, CS/HA nanocomposite coatings were biocompatible and, in particular, CS/10 wt.% HA composition significantly promoted human mesenchymal stem cells (hMSCs) proliferation. In particular, these results demonstrate that the treatment strategy used during the bioprocess was able to improve in vitro properties enough to meet the clinical performance. Indeed, it is predicted that the dense and crack-free CS/HA nanocomposite coatings suggest good potential application as dental implants.

## 1. Introduction

Titanium (Ti)-based alloys, especially Ti6Al4V, having a high strength-to-weight ratio, low density, excellent corrosion resistance, low cytotoxicity, biocompatibility, and good mechanical properties, constitute one of the most used materials for biomedical applications [1,2]. The formation of an oxide layer on Ti decreased the dissolution rate of Ti implants in the biological environment. However, there is concern about the interaction of Ti-based implants with bone tissue after implantation as the oxide layer could not promote the formation of a hydroxyapatite layer and the bone could not directly bond to Ti6Al4V implants. Moreover, previous studies have reported high levels of Ti ions in the area of the Ti-based implants [2]. In recent years, many surface treatments and coatings have been expanded to create interactions between Ti implants and the surrounding bone [3,4]. Hydroxyapatite (HA: Ca_10_(PO_4_)_6_(OH)_2_) coating as a principal inorganic element of natural bone can promote the formation of bone-like biological apatite on the implant surface [5]. Despite the advantages of HA for orthopedics and dental implant applications [6], its brittleness and low fracture toughness have been well-known as the main weaknesses of HA and have constrained its clinical applications [7,8]. Also, from the surgery point of view, the main problem of the use of HA is to apply them and keep them in place after implantation [5]. Consequently, mixing HA with a natural polymeric matrix, such as chitosan and silk fibroin, could improve the biomechanical properties [5,9] promote bone formation [10], and immobilize HA in a polymeric matrix [5]. Between them, chitosan (CS) as a natural copolymer of N-acetyl-glucosamine and N-glucosamine units could be a promising candidate due to its chemical similarity to biological molecules, tissue compatibility, bio-resorbability, antibacterial activity, bioactivity, biodegradation, non-toxicity to humans, and capability of chemical modifications [9,11,12,13]. CS/HA-based biomaterials have been extensively applied for bone substitutes, tissue engineering scaffolds and bone cement [14,15,16]. Different approaches have been established to make a ceramic or polymeric based coatings on the surface of metals or alloys [17,18,19,20]. It was reported that Ti6Al4V substrate was modified by a chitosan film to promote the nucleation of HA/Ag composite coatings deposited by the thermal substrate method [21]. The HA/Ag/CS coatings showed better cohesion HA crystals, adhesive strength to the substrate surface and antibacterial activity. Furthermore, Sorkhi et al. [22] reported the preparation of hydroxyapatite/chitosan-based nanocomposite coatings with different HA content using electrophoretic deposition (EPD). In particular, it was demonstrated that the presence of higher HA content induces the formation of some pores and crack. Similarly, HA/CS composite coatings with higher HA content prepared by micro-arc oxidation and dip-coating methods presented a porous micro-nano structure. As the loaded CS amount was increased, the antibacterial property enhanced [23]. However, the antibacterial properties may be improved by loading of specific compounds (i.e., antibiotics, antimicrobial peptides, etc) in order to obtain a local release able to inhibit bacteria growth and prevent the biofilm formation during in vivo implantation [24]. Among these approaches, the sol-gel technology provides attractive advantages, such as uniformity of composition, homogeneity of thin films, low cost, precise stoichiometry, and low sintering temperature [25,26,27]. Despite the wide application of HA nanopowders in the synthesis of HA-based composites [28,29], in-situ synthesis of HA nanoparticles by sol-gel method in the presence of the polymer solution is revealed to be a creating strategy for the attainment of homogeneous composite materials having higher degree of phase interaction among polymeric matrix and HA [30,31,32]. Results demonstrated that the incorporation of HA nanopowders higher than 30 wt.% in the CS/HA composites could lead to increased agglomeration, a lack of control of the molecular structure, and decreased bioactivity and mechanical properties [9,33]. However, the limited studies have focused on the properties of CS/HA nanocomposite coatings prepared by the in-situ sol-gel method. Otherwise, many studies reported the use of neat chitosan and its composites as a coating of dental implants [34,35] because it allows to improve the biological, morphological, and mechanical surface properties. In the case of mechanical properties, the chitosan may change the elastic modulus, thus reducing the discrepancy between the implant surface and alveolar bone and decreasing the areas of stress concentration [36]. The good adhesion strength between the coating and the Ti substrate provides the successful implantation and long-term stability of the coated implant as a considerable characteristic to access the mechanical integrity of the coating system [20]. For instance, Wang et al. [37] developed a CS/HA composite coating with different concentrations of CS on Ti implants and revealed a significant promotion of the interface bonding strength due to the interactions between HA, CS, and substrate. Also, factors such as chemical composition, surface morphology, surface energy, hydrophobicity, and surface roughness of CS/HA coatings would affect the cell response, the biocompatibility, and mechanical properties of implants in clinical use. In this way, this study aims to in-situ synthesize CS/HA nanocomposite coatings (HA content to 60 wt.%) on alkali-treated Ti6Al4V substrate via sol-gel process to obtain the crack-free coatings without any imperfection and pores. Indeed, the influence of HA concentrations (10, 50, and 60 wt.%) on the morphology, hydrophilicity, adhesion strength and roughness of CS/HA coatings were investigated. In particular, the results demonstrated that the combination of sol-gel method and alkali-treatment allows to obtain important improvements depending on the material composition. Finally, the effect on human mesenchymal stem cells (hMSCs) and in vitro bioactivity evaluation were evaluated. Here, the results showed statistically significant improvements in short term in vitro biological properties. This achievement will allow for the selection of a treatment that has the potential to meet the clinical performance requirements not currently being met for dental implant applications.

## 2. Materials and Methods

### 2.1. Materials

The chemicals used were chitosan (molecular weight ca. 300 kDa, Sigma-Aldrich, Milano, Italy), calcium nitrate tetrahydrate (Ca(NO₃)₂·4H₂O, Sigma-Aldrich, Milano, Italy), di-ammonium hydrogen phosphate ((NH4)_2_HPO_4_, Sigma-Aldrich, Milano, Italy), sodium hydroxide (NaOH, Sigma-Aldrich, Milano, Italy), distilled water (dH_2_O), and acetic acid (CH_3_COOH, Sigma-Aldrich, Milano, Italy).

### 2.2. In-Situ Synthesis of Chitosan/Hydroxyapatite (CS/HA) Coating

**Scheme 1:**
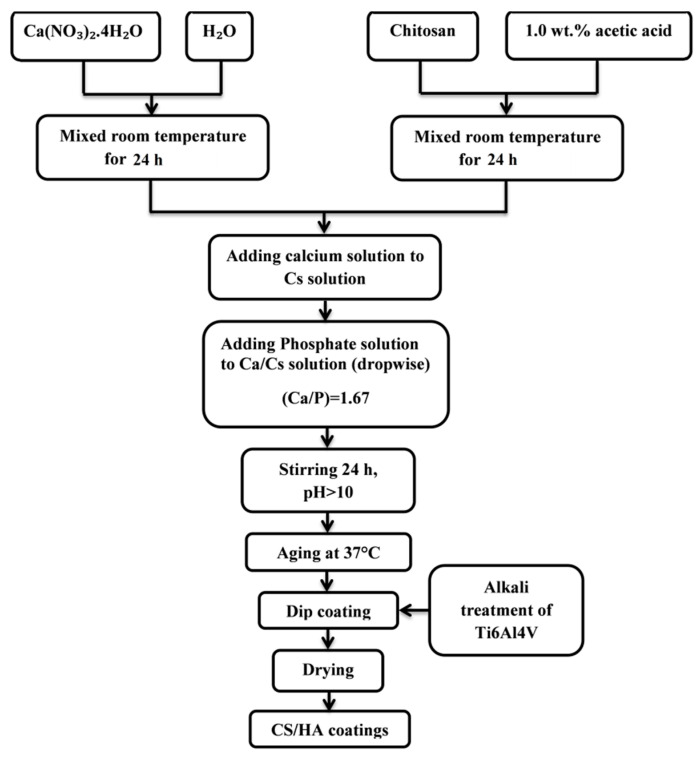
Flowchart of CS/HA coating by sol-gel process.

CS/HA coatings containing different amount of HA, 10, 50 and 60 wt %, were synthesized by sol-gel technology. Initially, 10 wt.% chitosan solution in 1.0 wt.% acetic acid was prepared after 60 min stirring at 50 °C. The solution was stirred for 24 h at 25 °C. For the in situ synthesis of HA, calcium nitrate tetrahydrate (Ca(NO₃)₂·4H₂O) and di-ammonium hydrogen phosphate ((NH_4_)_2_HPO_4_) were separately dissolved in distilled water (dH_2_O) and stirred for 24 h at 25 °C. Subsequently, Ca-based solution was added to the CS solution and the mixture was stirred for 4 h. Then, the phosphate solution was introduced drop by drop to Ca/CS solution and stirred for 24 h. At last, sodium hydroxide solution (1 M, NaOH) was used to adjust pH about 10. The amount of Ca, P and CS solutions were modified to have the composite with the composition of 10, 50, and 60 wt.% HA in CS matrix. Before coating process, Ti6Al4V substrates with the dimension of 20 mm × 10 mm × 2 mm were mechanically polished with 240–1000 grit-sized SiC papers and ultrasonically cleaned in acetone for 30 min. The alkali treatment of Ti6Al4V substrate was executed by incubating the samples in NaOH (5 M) solution at 60 °C for 48 h at 27% RH (relative humidity). Then, the specimens were rinsed with dH_2_O and dried at 37 °C for 24 h in air atmosphere. The coating process was applied using a simple dip-coating process at 25 °C. In this regard, the alkali-treated Ti6Al4V substrates were dipped vertically into CS/HA solution and pulled out at a constant speed of 60 (mm/min) and then dried at 37 °C in air for 2 h at 27% RH. This step repeated two times to obtain a suitable thickness. Finally, the coated substrates were treated at 37 °C for 48 h. A schematic flow chart of processing is reported below (Scheme 1).

### 2.3. Material Characterization

Phase composition analysis and microstructural characteristics were performed by X-Ray Diffraction (XRD, Philips X’Pert-MPD system (Eindhoven, The Netherlands), CuKα radiation (λ = 0.154 nm, 40 kV, 40 mA, between 10 to 60° on a 2θ scale in steps of 0.02 intervals with a counting time of 2 s at each step)) and scanning electron microscopy (SEM: Quanta 200, FEI Europe, Eindhoven, The Netherlands, different magnifications (500–20,000×)), respectively. For SEM analysis, the materials were coated with Au in a sputter coater (Coxem) up to a thickness of around 20 nm. Moreover, the HA nanocrystals size in CS/HA nano-composites was evaluated by using the Scherrer’s formula (Equation (1)) [38]:

(1) β=0.89λtcosθ

where *β* represents the peak width at half-maximum intensity of the reflection, *λ* is the X-ray wavelength for CuKα (*λ* = 0.15418 nm), *θ* is the Bragg diffraction angle, and t is the crystallite size in nanometers. Moreover, the HA crystallinity was calculated according to Equation (2) [39]:

(2) $$X_{c}=1-\left(V_{112/300}/I_{300}\right)$$

where *X_c_*, $V_{112/300},$ and $I_{300}$ are the degree of crystallinity, the intensity of the hollow between (112) and (300) reflections and the intensity of (300) reflection of hydroxyapatite, respectively.

Fourier transform infrared (FTIR) spectroscopy (Thermo Nicolet Avatar, Jersey City, NJ, USA) was applied to determine different functional groups. The hydrophilicity of the Ti6Al4V substrate before and after coating process was analyzed by water contact angle measurements in air, which were carried out using a drop shape analysis method (CAM-PLUS; TANTEC, Schaumburg, IL, USA). The measurements were performed by the sessile drop method at 25 °C and 27% RH. Four replicas of each sample were used and three drops of approximately 10 μL of deionized water were dropped onto three different points of each sample surfaces, and the static contact angle was measured for each drop. The adhesion strength between the coatings and substrate was determined according to standard ISO 13779-4 guideline [40]. For this test, the CS/HA coated disks (diameter and thickness of 25 and 1 mm), epoxy adhesive (RapidChem EA2003/H406, New Taipei City, Taiwan) and the universal tensile testing machine (Hounsfield, H5KS, Redhill, United Kingdom) at a fixed speed of 1 mm/min were used. The adhesion strength of the coated samples was computed by dividing the load, at the failure, by the original cross-sectional area; the analysis was performed in triplicate for each sample. Furthermore, the surface macroscopic topography was studied using laser profilometer (NCLP-Taicaan^®^ Technologies-Southampton, Southampton, United Kingdom) at 3 mm × 3 mm scans to determine the surface roughness and data analysis was performed using gwyddion Software (version 2.56, Czech Metrology Institute, Jihlava, Czech Republic).

In vitro bioactive behavior of the CS/HA coatings was assessed in simulated body fluid (SBF) solution which prepared according to Kokubo and Takadama’s protocol [41]. After day 21 of soaking, the materials were taken out from SBF, cleaned with dH_2_O and dried at 37 °C and 27% RH for 24 h. The surface morphology was examined using SEM microscopy. Moreover, the Ca and P ions concentrations after the incubation of samples were determined using the ICP-OES technique (Optima 7300DV, Perkin Elmer, Waltham, MA, USA).

### 2.4. In Vitro Cell Culture

The influence of CS/HA coatings at different compositions on cell proliferation was investigated using human Mesenchymal Stem cells (hMSC) obtained from LONZA (Milan, Italy). The cells were growth in alpha-MEM (Eagle’s alpha Minimum Essential Medium) supplemented with 10% FBS (Fetal Bovine Serum), antibiotic solution (streptomycin 100 µg/mL and penicillin 100 U/mL, Sigma Aldrich, Milano, Italy) and 2 mM L-glutamine at 37 °C and 5% CO_2_. The hMSCs were seeded at a density of 1 × 10^4^ cells/mL on the material surface and cultured for 1, 3, and 7 days. The cell proliferation was investigated using Alamar blueTM assay (AbD Serotec, Milano, Italy). This assay allows to evaluate the metabolic activity of the live cells and indirectly the cell viability [42]. In this case, the seeded substrates were incubated for 4 h at 37 °C, 5% CO_2_ with 500 µL of Alamar Blue^TM^ diluted 1:10 in DMEM without phenol red. Then, 200 µL of this solution was transferred into a 96-well plate for colorimetric analysis at wavelengths of 570 and 600 nm by using a spectrophotometer (Victor X3, Perkin Elmer, Milano, Italy). The experiment was performed in triplicate for three times.

Moreover, the cell-material interaction was studied using SEM microscopy after 3 days of culture. In this regard, the cells seeded on material surface were fixed using 2.5% glutaraldehyde solution for 2h and were dehydrated using graded ethanol solutions (25%, 50%, 75%, 90%, and 100%).

### 2.5. Statistical Analysis

All data were reported in mean ± standard deviation (SD) and statistical significance was measured by performing one-way ANOVA analysis using GraphPad Prism 8.2.1 (GraphPad Software, San Diego, CA, USA). *P* values < 0.05 were taken significant.

## 3. Results

### 3.1. Alkali-Treatment of Ti6Al4V Substrates

**Figure 1:**
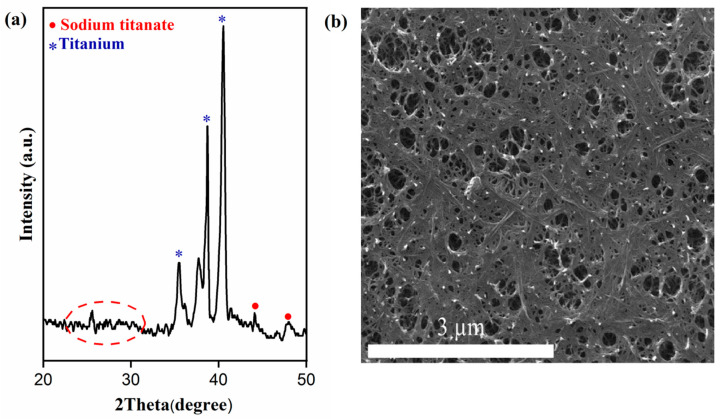
(**a**) XRD pattern and (**b**) SEM image of Ti6Al4V substrates after alkali-treatment approach.

Before nanocomposite coating, in order to mechanically interlock the coating to the substrate, alkali-treatment was performed. The effect of alkali-treatment on Ti6Al4V substrate was determined using XRD and SEM techniques. The XRD result and surface morphology of the Ti6Al4V substrate are shown in Figure 1a,b, respectively. According to Figure 1a, the XRD pattern of alkali treated Ti6Al4V indicated two peaks of sodium titanate at 2θ = 44 and 48°. In addition, there were broad diffraction peaks at 2θ = 23–30°, relating to the sodium titanate formed on the Ti6Al4V substrate. It was similarly reported [4] that, during the alkali treatment, the superficial TiO_2_ layer partially dissolved into the alkaline solution due to the attack by hydroxyl groups, according to the following Reaction (R1):

(R1) $${\mathrm{TiO}}_{2}+\mathrm{NaOH}\;\to {\mathrm{HTiO}}_{3}^{-}+{\mathrm{Na}}^{+}$$

The titanium also reacted simultaneously with NaOH solution, and these reaction mechanisms were as follows:

(R2) $$\mathrm{Ti}+3{\mathrm{OH}}^{-}\to \mathrm{Ti}{\left(\mathrm{OH}\right)}_{3}^{+}+4e^{-}$$

(R3) Ti(OH)3++e−→TiO2·H2O+12H2↑

(R4) $$\mathrm{Ti}{\left(\mathrm{OH}\right)}_{3}^{+}+{\mathrm{OH}}^{-}↔\mathrm{Ti}{\left(\mathrm{OH}\right)}_{4}$$

A further hydroxyl attack on the hydrated TiO_2_ produces negatively charged hydrates on the surface of the substrates as follows:

(R5) $${\mathrm{TiO}}_{2}\cdot H_{2}O+{\mathrm{OH}}^{-}↔{\mathrm{HTiO}}_{3}^{-}\cdot {\mathrm{nH}}_{2}O$$

Finally, titanate hydroxide $\left({\mathrm{HTiO}}_{3}^{-}\cdot {\mathrm{nH}}_{2}O\right)$ reacted with Na^+^ ions and porous shaped sodium titanate hydrogel was formed on Ti6Al4V surface [43].

SEM image of alkali-treatment samples (Figure 1b) also indicated the presence of a porous structure which may be due to the corrosive attack of the hydroxyl groups in NaOH solution [4]. The presence of the nanoporous structure with an average diameter of about 100 to 300 nm could remarkably increase the surface area of substrate, which provided more nucleation sites for the next coating anchored to the substrate. Previous studies reported that this porous structure could enhance the hydrophilicity and roughness of substrates which could promote the adhesion strength between the coating and Ti6Al4V substrate [4,11].

### 3.2. Characterization of CS/HA Coatings

**Figure 2:**
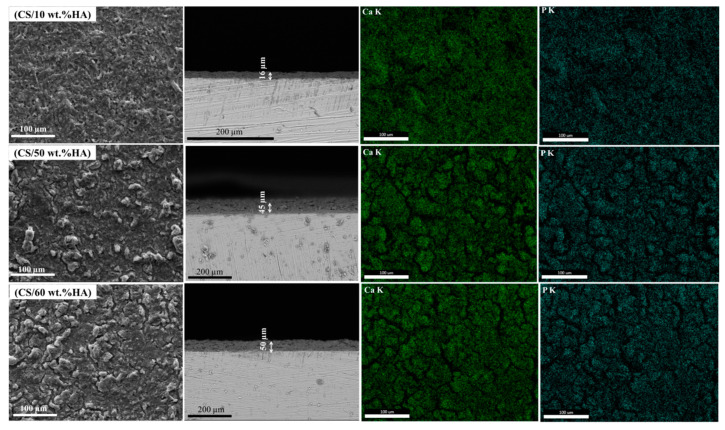
Surface morphology in cross section and EDS mapping of CS-HA coatings with 10, 50 and 60 wt.% of HA.

SEM images and EDS mapping of CS/HA coatings with different HA content (10, 50 and 60 wt.%) are showed in Figure 2. The SEM images represented the formation of crack-free coatings without any imperfections, especially at CS/10 wt.% HA coating. In addition, the cross-section images also revealed the formation of dense and uniform coatings with a thickness in the range of 15–50 µm, absence of any delamination or mismatch at the coatings/Ti6Al4V substrate interface which could be due to the alkali-treatment of the substrates. In addition, EDS mapping-analysis confirmed the homogenous distribution of HA, specifically at CS/10 wt.% HA coating. Meanwhile, in the samples with higher HA amount (CS/50 wt.% HA and CS/60 wt.% HA coatings) some clusters of HA nanoparticles were observed. Furthermore, the size/shape of HA crystalline depends on the [Ca^2+^] and [PO_4_^3−^] ion concentration or the rate of crystalline growth [44]. Indeed, SEM images and EDS mapping of CS/HA coating consisting of 50 and 60 wt.% HA indicated that higher [Ca^2+^] and [PO_4_^3−^] concentration accelerated the crystalline growth rate and agglomeration. Moreover, with increasing HA content, the surface morphology of CS/HA coatings changed due to variation of nucleation and HA crystals growth with an improvement of surface roughness. In the homogeneous and smooth CS/10 wt.% HA coating, HA crystals grew slowly with uniform distribution. In contrary, incorporation in the coatings of HA at higher amount (50 and 60 wt.% of HA) resulted in the formation of much denser and roughness surface. According to the EDS analysis of CS/HA coatings (Supplementary Figure S1), HA with Ca/P ratio around 1.74 (CS/10wt.% HA), 1.63 (CS/50 wt.% HA), and 1.68 (CS/60 wt.% HA) was obtained in the composite material.

**Figure 3:**
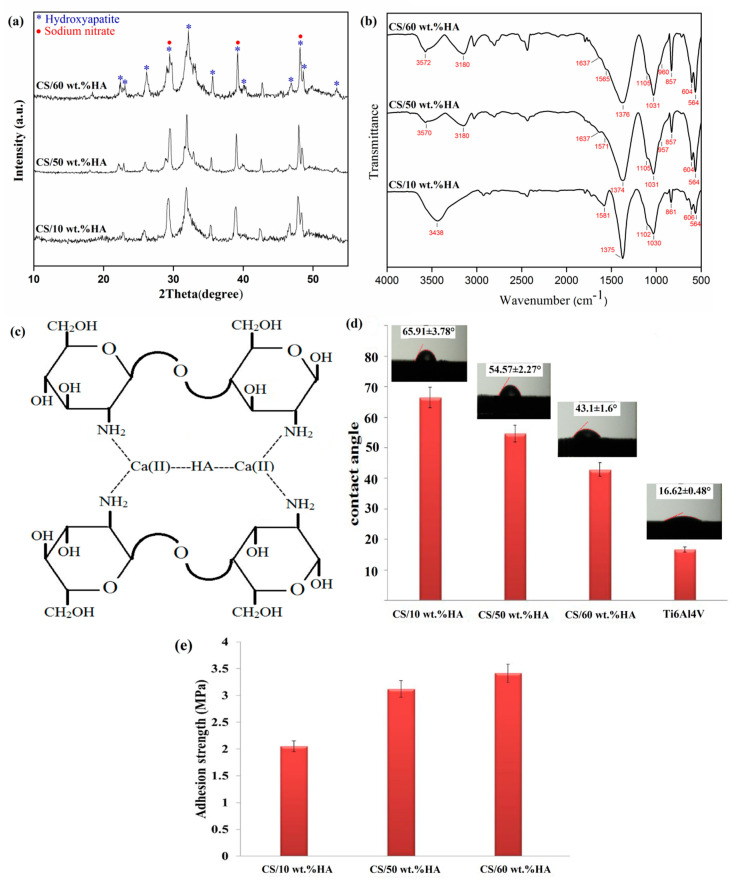
(**a**) XRD patterns and (**b**) FTIR spectra of CS/HA with 10, 50 and 60 wt.% of HA (**c**) Interaction between Ca^2+^ of HA and –NH_2_ of chitosan (**d**) Contact angle between the water drop and the sample surfaces (**e**) Adhesion strength of the CS/HA coatings.

To confirm the presence of chitosan and HA components in the coatings, XRD and FTIR analyses were performed. XRD patterns of CS/HA composite powders (Figure 3a) confirmed the presence of HA characteristic peaks, matched with a JSPDS card number of 09-432, but the XRD patterns showed the presence of sodium nitrate, consequently composites washed several times in dH_2_O to remove sodium nitrate residues. Moreover, the intensity of the HA diffraction peaks enhanced with increasing HA content. By Equation (2), the crystallinity of HA in the CS/HA with 10, 50 and 60 wt.% of HA is determined to be about 23%, 28%, and 33%, respectively. However, the HA crystallization in the nanocomposites was not significant which could be related to the synthesis process at the low temperature. However, it has been demonstrated that HA with low crystallinity has higher levels of bioactivity [30]. Moreover, the crystalline size of HA in nanocomposites consisting of 10, 50 and 60 wt.% HA was estimated about 33, 50 and 53 nm, respectively. Indeed, the HA crystal size increased with decreasing chitosan content. This might be related to higher [Ca^2+^] and [PO_4_^3−^] values in chitosan solution during the synthesis of CS/HA composite. Moreover, it was probably due to the presence of chitosan which delayed the crystallization process. In the CS/60 wt.% HA material, HA crystals grew quickly and the crystallinity increased due to the lower chitosan amount which led to increase the size of HA crystals and finally the rate of crystalline growth.

FTIR spectra of nanocomposites (Figure 3b) confirmed the presence of chitosan in the combination with HA. According to this figure, the ionic agent of phosphate (PO_4_^3−^) bands at around 564, 606 (bending vibration bands) and 1030–1105 cm^−1^ (stretching vibration bands) were observed [45]. Costescu et al. [46] demonstrated that the band at around 857 cm^−1^ was associated with ν2 vibrations of carbonate groups. Moreover, the appearance of bands at 1033, 1100, and 1375 cm^−1^ may be also associated to the stretching of C-O and CH_3_ wagging, respectively [47]. The broad band at 1030 cm^−1^ corresponded to the polymer and its interaction with the phosphate groups in composite [16]. The band at 1565 cm^−1^ was related to NH bending (amide II), which shifted to a higher wavenumber at 1571 cm^−1^ and 1581 cm^−1^ corresponding to the increasing of CS in composites [47]. FTIR results of CS/HA were in accordance with the infrared spectra reported by Xianmiao et al. [28]. Ca^2+^ ions with a coordination number of 7 was revealed on the terminated surface of HA crystals, which were strictly held in the structure. Therefore, there might be formed the coordination bond between the –NH_2_ of chitosan and Ca^2+^ of HA (Figure 3c). Moreover, the bands were related to the amine (1655 cm^−1^) and amide (1599 cm^−1^) in pure chitosan [15] shifted to lower wavenumbers in the CS/HA composite as a result of interaction between chitosan and HA, through hydrogen bonds between –NH_2_ and –OH as well as chelation between –NH_2_ and Ca^2+^.

It has been described that hydrophilic surfaces led to the enhancement of cell proliferation [48,49,50] and increasing of the ion-exchange behavior from the SBF solution and then enhanced the apatite growth [51]. Figure 3d shows the typical images of water drop and contact angle on the alkali-treated Ti6Al4V and the CS/HA coatings. There was a tendency to employ the alkali-treatment method to improve the substrate hydrophilicity. Indeed, the water contact angle of the alkali-treated Ti6Al4V surface was 16.62±0.48° that indicated high hydrophilicity of material surface due to the change of surface functional groups during the alkali-treatment. The formation of uniform CS/HA coatings without any mismatch at the coatings/Ti6Al4V interface (Figure 2) resulted in the alkali-treated substrate Ti6Al4V due to increasing hydrophilicity. However, the water contact angle of samples enhanced to 65.91 ± 3.78°, 54.57 ± 2.27° and 43.1 ± 1.6°, after surface treatment with CS/HA coatings containing 10, 50 and 60 wt.% HA, respectively. Increased hydrophilicity of nanocomposite coatings with increasing HA content might be due to the more existence of hydrophilic hydroxyl-terminated groups on surfaces. Moreover, the surface roughness of the CS/60 wt.% HA coating sample (Figure 2), induced an increasing of the surface coating hydrophilicity. Thus, water contact angle results showed that CS/60 wt.% HA coating provided the best hydrophilic properties. The improvement of adhesion strength between the coatings and the substrates seem to be the essential matter in long-term stability and successful implantation under physiological conditions [52]. The detachment of the coatings may occur due to the poor adhesion of the coatings. Figure 3e presents the adhesion strength of the CS/HA coatings. The adhesion strength of CS/HA coatings containing 10, 50 and 60 wt.% HA was 2.05 ± 0.08, 3.11 ± 0.16 and 3.42 ± 0.14 (MPa), respectively. Here, it was demonstrated that the alkali-treatment increased the adhesion strength between the coatings and Ti6Al4V substrate [4]. Furthermore, results showed that the adhesion strength of the CS/HA coated specimens could be improved by increasing HA, which was attributed to the polymer/particle adhesion due to chemical reactions and physical bonding [53]. Also, the improved adhesion strength of the coatings with increasing HA could be due to greater roughness and surface area (Figure 2), which enhance the strength bonding between substrate and coatings [54].

**Figure 4:**
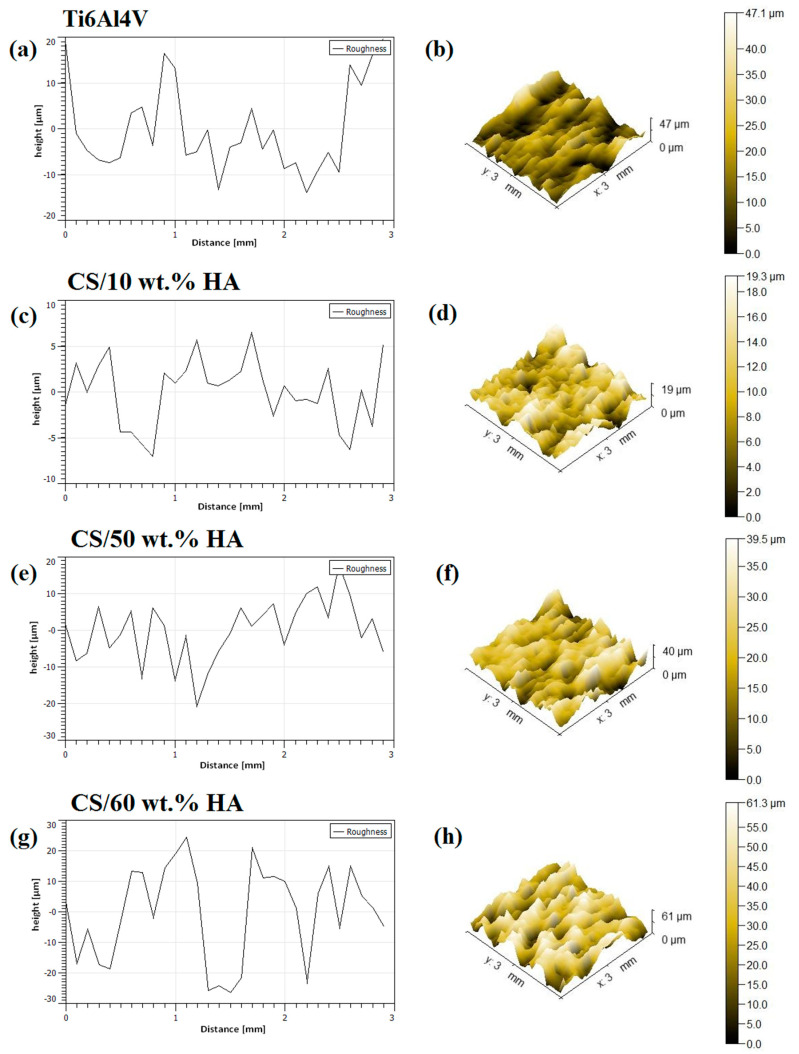
2D and 3D optical interferometry images revealing micro-waviness (**a**,**c**,**e**,**g**) and macro-waviness (**b**,**d**,**f**,**h**) features of alkali-treated Ti6Al4V surface, CS/HA coating surfaces consisting of various amounts of HA content.

The surface roughness is a well-known factor that determines the biological properties such as apatite layer deposition in SBF and cellular responses [23]. Based on the clinical results from retrieved implants, rough surface of implants can improve the ingrowth of soft and hard tissue into the materials, thereby making more biological anchorage to enhance the stability of the Ti-base implant [55]. The surface roughness of the alkali-treated Ti6Al4V substrate and CS/HA coatings was estimated using profilometer applying a laser sensor. The 2D and 3D optical interferometry images are showed in Figure 4. Results approved the presence of porous structure obtained during alkali-treatment according to enhance in the surface roughness (R_a_ = 8 ± 2 µm). This topography has promoted a good bonding among Ti6Al4V substrate and CS/HA coating. Compared to CS/10 wt.% HA coating, the CS/50 wt.% HA and CS/60 wt.% HA exhibited more surface roughness (CS/10 wt.% HA, R_a_ = 2.88 ± 1.5 µm; CS/50 wt.% HA, R_a_ = 6.67 ± 2.1 µm; CS/60 wt.% HA, R_a_ = 12.93 ± 4.5 µm). The surface roughness improvement of coated specimens with increasing HA content might be due to the agglomeration of HA in the chitosan matrix and the increased surface inhomogeneity. The surface roughness results were in agreement with the SEM results. Previous studies have reported the surface roughness of the coated implants was measured to be between 1 and 20 μm according to the R_a_ parameter [18,56].

### 3.3. In Vitro Bioactivity Assessment of CS/HA Coatings

**Figure 5:**
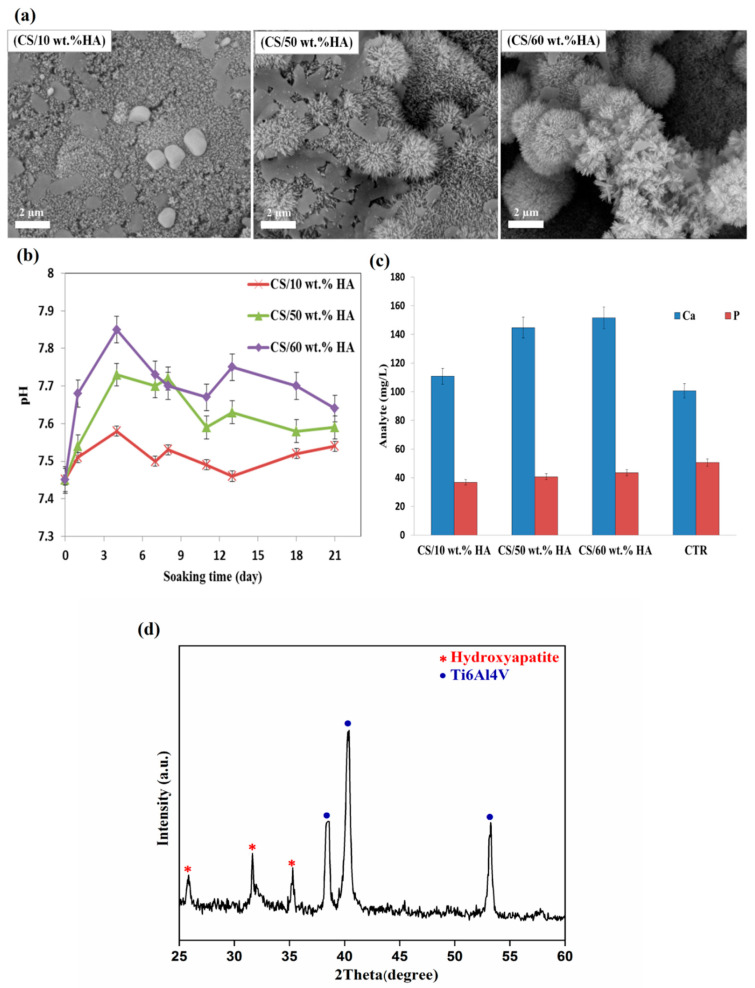
(**a**) SEM images of apatite deposited on the surface of CS-HA coatings immersed in SBF 1X for 21 days (**b**) pH of the SBF solution, after soaking the CS/HA coatings (**c**) Ca and P ions concentrations of the CS/HA coatings after 21 days soaking (**d**) XRD pattern of CS/60 wt.% HA coating after 21 days soaking.

The bioactivity potential in SBF solution has been regarded as a key evidence of bone-like apatite layer production on the material surface that is an essential condition to achieve direct bonding with living bone [57]. Figure 5a represents SEM images of CS/HA coatings at day 21 of incubation in SBF. All samples showed the nucleation of apatite particles on the surface of CS/HA coating. In particular, SEM images of CS/10 wt.% HA coating showed that surfaces were coated with formless apatite particles after 21days of soaking in SBF. A typical “cauliflower” morphology was distinctly observed for CS/50 wt.% HA and CS/60 wt.% HA samples after 21 days of incubation, in which each apatite particles included many needle-like crystals. The surface morphology for CS/60 wt.% HA coating indicated highly dense apatite deposition and growth compared to other samples. It appears that because of the increasing HA content in coating, more nucleation sites were offered to promote nuclei formation and crystallization of apatite layer. Indeed, the formation apatite-like layer may be modulated by altering the quantity of HA in the composite during the immersion in SBF [5]. Samandari et al. [58] found that nano-HA particles in the nanocomposite scaffolds played the primary role in apatite layer formation. According to Figure S2, the deposition consisted of Ca and P atoms with the ratio around 1.64 (CS/10 wt.% HA), 1.66 (CS/50 wt.% HA), and 1.61 (CS/60 wt.% HA), which were close to 1.67 of bone-like apatite. During SBF treatment, pH values of SBF solution were changed depending on the sample type (Figure 5b). The increasing rate of pH value depended on CS/HA composition. In composite with 10 wt.% HA, the pH values did not change remarkably. Increasing the amount of HA led to more pH changes. The pH value of SBF solution in all coated samples increased during the first five days of incubation, due to the exchange between hydrogen ions within the SBF solution and cations. Then, by increasing the soaking time for all samples a decreasing of pH values and the formation of bone-like apatite layer were observed [59]. Moreover, the variations of Ca and P ions concentrations during the SBF treatment of all samples for 21 days were reported in Figure 5c. The obtained results indicated that Ca ions concentration of the SBF solutions increased after day 21 of treatment. Conversely, phosphorus ions were consumed in the SBF solution and decreased gradually after 21 days soaking in all coated samples. The release of Ca^2+^ (Figure 5c) and the increased pH value of the SBF solution (Figure 5b) may be described by the Kokubo exchange mechanism for the formation and growth of the apatite on the surface coatings [41,60]. As reported in the previous studies, when the CS/HA coating was immersed in SBF solution, the coatings react with the SBF solution. In this way, dissolution and re-precipitation of coated specimens occur, leading to the formation of apatite [5,31]. Furthermore, the XRD pattern (Figure 5d) confirmed the apatite formation on the surface of CS/60 wt.% HA coating after biomimetic treatment for 21 days and it was also determined the low crystallization similar to the HA of natural bone. Finally, SEM images, XRD, ICP investigations and pH changes results suggested that the CS/HA coatings prepared by the in-situ sol-gel process were bioactive. The obtained results demonstrated that the presence of hydrophilic nanocomposite coating with higher HA content (CS/60 wt.% HA) in the SBF solution provided more nucleation sites and might accelerate the apatite mineralization and suggesting a good bioactivity [61]. It has been reported that the bioactive coatings could promote an attachment of bone to the implant and improved osseointegration between implant and host tissue [62,63]. These findings suggested that increased bond strength and accelerated osteoconductivity are very important aspects of CS/HA coated implant applications.

**Figure 6:**
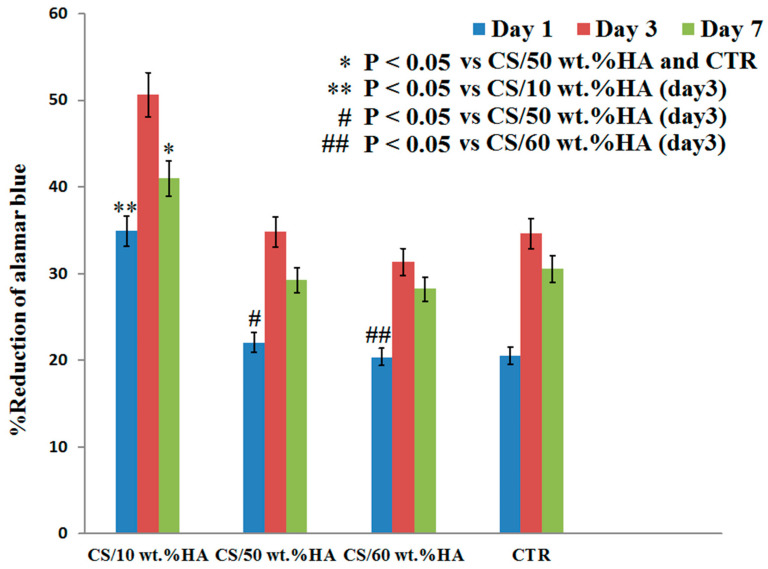
Alamar blue assay after 1, 3 and 7 days of cell culture. Data were normalized and compared (mean, SD, n = 3) using a one-way ANOVA, *P* < 0.05.

Furthermore, in vitro biological study was performed to evaluate the effect of surface properties on cell proliferation [64]. In particular, hMSCs proliferation was quantified after one, three, and seven days of culture on the CS/HA coatings, and the results are shown in Figure 6. Results demonstrated that the cell proliferation for all samples showed the same behavior of control (CTR) with the peak at day 3 of incubation time, suggesting that the CS/HA coated samples could support the hMSCs proliferation without any cytotoxic effect. In particular, according to the results, after day 1 of culture, the cell proliferation on all of the coating increased and, CS/10 wt.% HA coating presented the highest proliferation compared to other samples. By increasing HA content, there were no significant differences between CS/50 wt.% HA and CS/60 wt.% HA coatings (*P* > 0.05). A greater proliferation rate occurred between one and three days followed by a slow-down until 7 days. There was a significant difference in cell proliferation among the CS/HA coatings with 10, 50 and 60 wt.% of HA at one day compared to three days (*P* ˂ 0.05). Considerable differences were observed at day 3 for CS/10 wt.% HA vs CS/50 wt.% HA and control (CTR) (*P* ˂ 0.05). This behavior was similarly reported by Xianmiao et al. [28] who prepared hydroxyapatite/chitosan membranes for enhanced guided bone regeneration. They disclosed that the CS/HA membranes had no negative effect on the cell viability and possess good biocompatibility.

**Figure 7:**
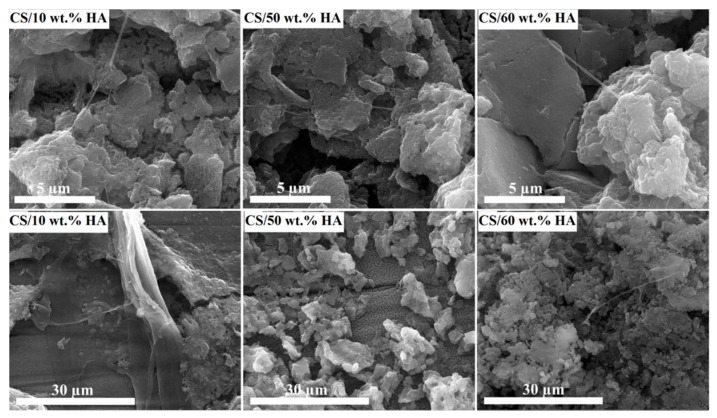
SEM micrographs of hMSCs on the CS/HA coatings at two different magnifications (5000× and 20,000×).

The SEM micrographs of CS/HA coatings after three days of culture are showed in Figure 7. Different surface morphology of CS/HA coating with varying roughness significantly has a significant effect on cell proliferation. As observed, cells interacted with all CS/HA coatings. In the presence of 10 wt.% HA, the cells showed a spread appearance on the coating surface. By increasing the amount of HA content, only fewer cells stretch and proliferate on the coating surface due to HA nanoparticles agglomeration. Based on Alamar blue reduction and SEM results, smooth and homogeneous CS/10wt.% HA coating morphology without the agglomeration of HA nanoparticles could promote cell proliferation compared to the rough CS/50 wt.% HA and the CS/60 wt.% HA coatings morphologies. The smooth and homogeneous surface coatings clearly induced better cell adhesion, viability, and proliferation than rough surfaces. This behavior may be attributed to the larger contact surface area between the cells and the coating [20,65]. Kamitakahara et al. [66] reported that the wide contact surface area is an important factor for cell proliferation and cells could proliferate easily. Mokabber et al. [20] indicated the smooth surface of calcium phosphate coatings resulted in better proliferation of the cells, whereas the rough calcium phosphate coatings restricted cell adhesion and consequently cell proliferation. According to the above points, a good cell proliferation in the CS/10 wt.% HA coating was a result of smooth and homogeneous morphology with wide contact surface area.

## 4. Conclusions

In this study, CS/HA coatings with 10, 50 and 60 wt.% of HA were synthesized on alkali treated Ti6Al4V substrate by in-situ sol-gel method and characterized regarding microstructure, contact angle degree, adhesion strength, roughness and in vitro bioactivity and biocompatibility. In particular, samples with less HA content (CS/10 wt.% HA) show homogeneous HA distribution and lower surface roughness with a good cellular response in terms of cell proliferation. Meanwhile, higher HA content (CS/50 wt.% HA and CS/60 wt.% HA) in the composite coating improves surface hydrophilicity, adhesion strength, and roughness. Furthermore, in vitro bioactivity evaluation indicated that the precipitation of the apatite improved with increasing HA in the coating due to more nucleation sites during SBF treatment for 21 days, demonstrating that CS/HA materials were not only biocompatible but also bioactive coatings. In this way, the obtained results demonstrated that the used treatment strategies are able to improve in vitro properties and bioprocesses and to meet the clinical performance of bioactive coatings for implant applications.

## Supplementary Materials

The following are available online at https://www.mdpi.com/1996-1944/13/17/3772/s1, Figure S1. EDS analysis of CS/HA coatings with 10, 50 and 60 wt.% of HA.; Figure S2. EDS analysis of apatite deposited on the surface of CS/HA coatings with 10, 50 and 60 wt.% of HA immersed in SBF 1X for 21 days.
