# Supplementary material for: In-Situ Synthesis and Characterization of Chitosan/Hydroxyapatite Nanocomposite Coatings to Improve the Bioactive Properties of Ti6Al4V Substrates
Source: Materials (Basel). 2020 Aug 26;13(17):3772. doi: 10.3390/ma13173772 (PMC7503881; doi:10.3390/ma13173772)
Supplement: Supplementary file 1 [file materials-13-03772-s001.pdf]

materials

MDPI

In-situ synthesis and characterization of Chitosan/Hydroxyapatite nanocomposite coatings to improve the bioactive properties of Ti6Al4V substrates

Zahra Ansari[1], Mahdi Kalantar[1*], Alessandra Soriente[2], Ines Fasolino[2], Mahshid Kharaziha[3], Luigi Ambrosio[2], Maria Grazia Raucci[2*]

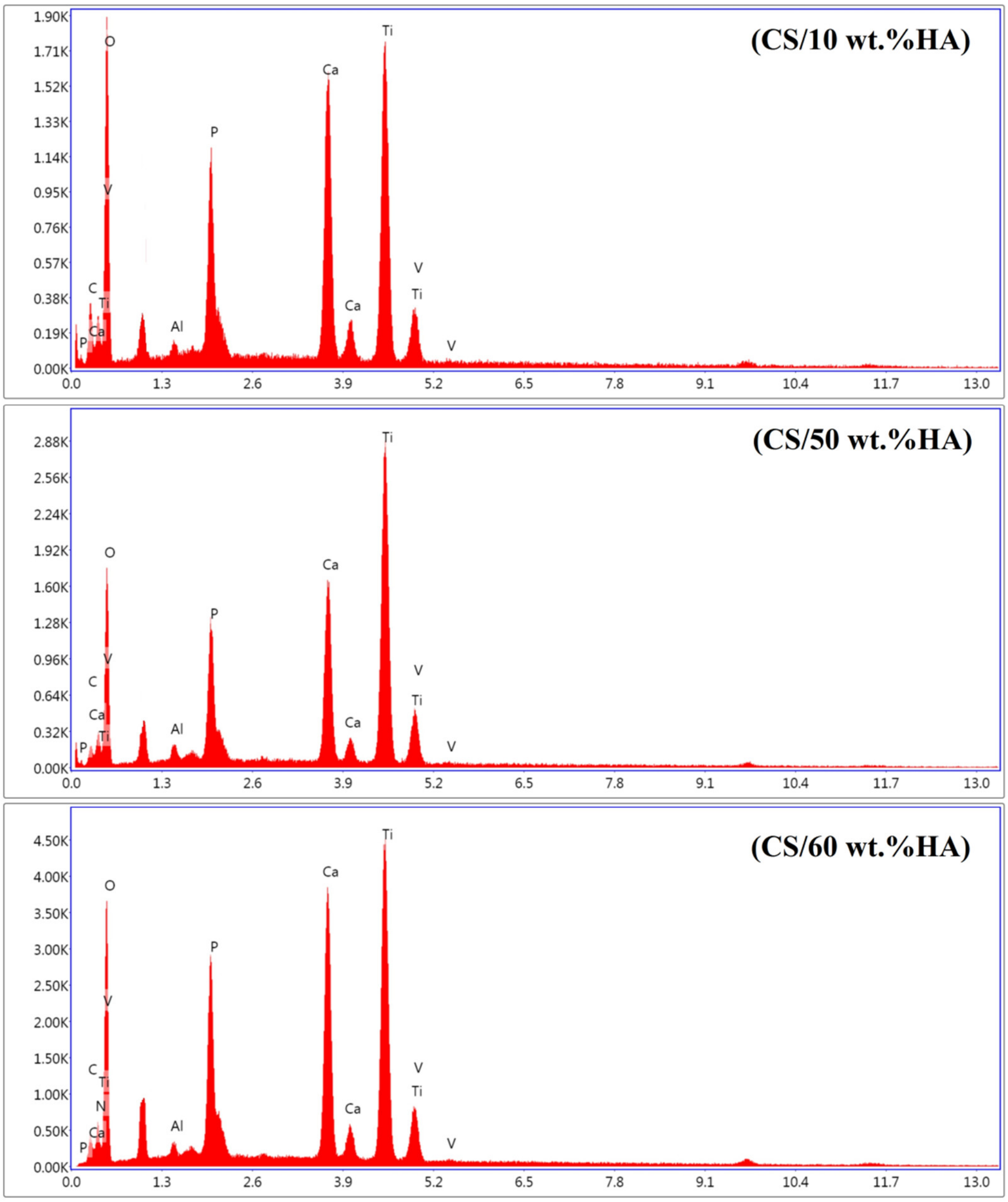

Figure 1. EDS analysis of CS/HA coatings with 10, 50 and 60 wt.% of HA.

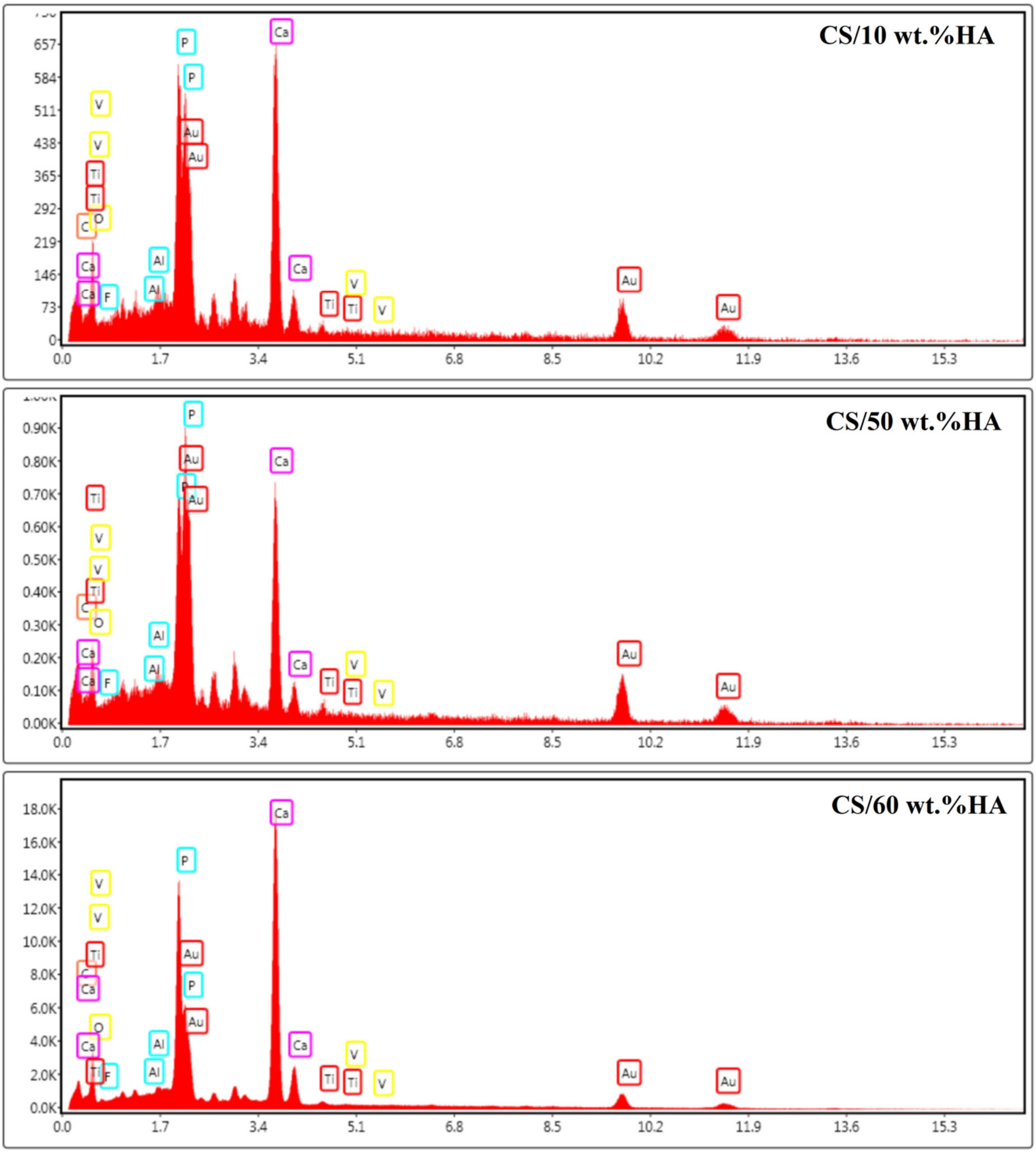

Figure S2. EDS analysis of apatite deposited on the surface of CS/HA coatings with 10, 50 and 60 wt.% of HA immersed in SBF 1X for 21 days.
